# Peccavimus

**Published:** 1868-08-01

**Authors:** 


### *Peccavimus.*

The even tenor of the Journal's way, and the harmony which should every where prevail in its columns, was broken and jarred in the last number by the introduction of a communication bearing upon medical ethics, politics and civil war. We know nothing (and care less), about the allegations involving individuals therein made. Our pigeon-holes are crammed with papers communicating instances of violations of the "Code," by men high in professional rank, and case after case, either of mistaken diagnosis, or willful fraud, by practitioners whose names are in the odor of medical sanctity, but we forbear assuming this kind of scavenger work. We have no stomach for "policing the camp." The scattered rattle of the rifles of the frightened pickets disturbs the army more than all the batteries of the enemy. Our big guns are aimed at Ignorance, not at individuals, and we propose to fight it out on *that* line to the end of our editorial life. If a little wholesome discipline is occasionally needed by *noisy* subalterns or *brigadiers*, the Journal will conduct its own *courts-martial*. Meanwhile, "Dress up on the right!"

